# Tannic Acid-Enhanced Gelatin-Based Composite Hydrogel as a Candidate for Canine Periodontal Regeneration

**DOI:** 10.3390/gels11080650

**Published:** 2025-08-15

**Authors:** Laura C. Pinho, Marta Ferreira, Angélica Graça, Joana Marto, Bruno Colaço, Maria Helena Fernandes, Catarina Santos

**Affiliations:** 1 BoneLab, Faculdade de Medicina Dentária, Universidade do Porto, Rua Dr. Manuel Pereira da Silva, 4200-393 Porto, Portugal; al61180@alunos.utad.pt; 2 LAQV/REQUIMTE, Faculdade de Medicina Dentária, Universidade do Porto, Rua Dr. Manuel Pereira da Silva, 4200-393 Porto, Portugal; 3 Centre for Research and Technology of Agro-Environmental and Biological Sciences, CITAB, Inov4Agro, University of Trás-os-Montes and Alto Douro, UTAD, Quinta de Prados, 5000-801 Vila Real, Portugal; bcolaco@utad.pt; 4 CQE, IMS, Instituto Superior Técnico, University of Lisbon, 1049-001 Lisbon, Portugal; catarina.santos@estsetubal.ips.pt; 5 Instituto Politécnico de Setúbal, EST Setúbal, Campus IPS-Estefanilha, 2910-761 Setúbal, Portugal; marta.silva.ferreira@estudantes.ips.pt; 6 Research Institute for Medicines (iMed.ULisboa), Faculdade de Farmácia, Universidade de Lisboa, Avenida Professor Gama Pinto, 1649-003 Lisboa, Portugal; angelicagraca@campus.ul.pt (A.G.); jmmarto@ff.ulisboa.pt (J.M.); 7 CECAV—Veterinary and Animal Research Centre, AL4AnimalS, Universidade de Trás-os-Montes e Alto Douro, UTAD, Quinta de Prados, 5000-801 Vila Real, Portugal

**Keywords:** gelatin-based hydrogel, canine periodontal ligament, alveolar bone, hydroxyapatite nanoparticles, graphene oxide, guided tissue regeneration

## Abstract

Periodontal disease in dogs leads to progressive bone loss and adversely impacts overall health. However, cost-effective regenerative strategies are still limited in veterinary practice. This study aimed to develop and evaluate a novel tannic acid (TA)–gelatin-based hydrogel (Gel), incorporating graphene oxide (GO) and hydroxyapatite nanoparticles (HA), as a potential barrier material for guided tissue regeneration (GTR) applications. The hydrogels—Gel, Gel-GO, Gel-HA, and Gel-GO-HA—were characterized for chemical structure, molecular interactions, surface morphology, nanoparticle dispersion, and tensile strength. Cytotoxicity was assessed using L929 fibroblasts (ISO 10993-5), while cell viability/proliferation, morphology, and alkaline phosphatase (ALP) production were evaluated using canine periodontal ligament-derived cells. Results show that crosslinking with tannic acid enhanced the incorporation of graphene oxide and hydroxyapatite nanoparticles via hydrogen bonding into TA–gelatin-based hydrogels. This combination increased surface roughness, reduced degradation rate, and enabled shape memory behavior, critical for guided tissue regeneration (GTR) membranes. The extracts from Gel-HA-GO showed that cytotoxicity was both time- and concentration-dependent in L929 fibroblasts, whereas enhanced cell proliferation and increased ALP production were observed in cultures derived from canine periodontal ligament cells. These findings suggest that TA–gelatin-based hydrogels incorporating GO and HA demonstrated favorable mechanical and physicochemical properties, biocompatibility, and osteogenic potential. These attributes suggest their viability as a promising composite for the development of innovative GTR strategies to address periodontal tissue loss in veterinary medicine.

## 1. Introduction

Bone loss due to periodontal disease (PD) profoundly impacts the health and quality of life of dogs. As the disease progresses from mild to severe periodontitis, it is marked by increased gingival inflammation and recession, loss of periodontal attachment, and ultimately, tooth loss resulting from alveolar bone resorption. This progression allows bacteria and degradation products to potentially enter the bloodstream, which may contribute to the development of a systemic condition. Periodontal therapy is designed to manage and treat periodontal disease by controlling infection and preventing further tissue destruction. Initial treatment typically includes scaling and root planning, which are essential for the removal of plaque and reduction in inflammation. When inflammation or increased pocket depth persists despite initial therapy, local antibiotic application and surgical interventions such as flap surgery, bone grafting, or guided tissue regeneration may be necessary to restore the structural integrity of the periodontal supporting tissues [1,2]. Given this, there is a growing demand for treatments that address more than just managing the symptoms. Although tissue engineering has yielded successful guided bone regeneration (GBR) solutions for addressing periodontal bone loss in humans [3,4], comparable treatments for dogs are still limited. Despite ongoing research efforts [5,6], there remains a lack of cost-effective options for veterinary applications, as well as a shortage of clinical trials in dogs with naturally occurring PD to evaluate the efficacy of these treatments [7].

Biodegradable membranes, composed of natural and/or synthetic polymers, can be tailored to meet specific criteria. GTR membranes are designed to separate bone defects from surrounding epithelial tissues (cell-occlusiveness), creating a barrier effect that supports tissue regeneration while allowing the exchange of essential nutrients [8]. A significant advantage of biodegradable membranes is the elimination of the need for a second surgical intervention for removal, which reduces healthcare costs, minimizes patient discomfort, and lowers the risk of postoperative complications [3]. However, commercially available collagen-based membranes have been associated with inconsistent structural stability and rapid degradation under physiological conditions [9,10], emphasizing the necessity for the development of novel polymer composites with tailored characteristics for membrane production.

Gelatin, a natural polymer derived from the partial hydrolysis of collagen, is known for its biocompatibility, ease of functionalization, and cost-effectiveness [11]. As a hydrolyzed form of collagen, gelatin features arginine–glycine–aspartic acid (RGD) sequences that promote cell adhesion and proliferation [12]. Its molecular structure allows it to serve as both an encapsulation material and a delivery platform for therapeutic agents, facilitating sustained and localized drug release [11]. Gelatin is categorized into type A and type B, depending on its preparation method, with each type tailored for specific applications according to its isoelectric properties, which enhance the integration of electrically charged therapeutic agents. Type A gelatin, produced through an acidic process, has an acidic pH, making it positively charged at physiological pH, and is well-suited for delivering negatively charged acidic bioactive agents. In contrast, type B gelatin, prepared via alkaline pre-treatment, exhibits a basic pH, is negatively charged at physiological pH, and is more effective for carrying positively charged basic bioactive compounds [13]. Despite these advantages, gelatin is characterized by poor mechanical strength and rapid degradation. These limitations can be addressed through chemical [14] or enzymatic cross-linking [7], as well as by combining with other reinforcing agents to enhance its mechanical stability, regulate its degradation rate, and improve the overall performance of the hydrogel [15].

Graphene oxide (GO), a derivative of graphene, has gained significant interest in the field of biomedical applications. With its carboxyl, hydroxyl, and epoxy groups, GO exhibits enhanced amphiphilicity and reactivity compared to graphene [16,17]. The carboxyl groups confer a negative charge to GO, while hydroxyl groups facilitate hydrogen bonding, improving its compatibility with various materials [16]. Additionally, GO is also characterized by its good mechanical properties, including tensile strength [18]. As a result of its physicochemical and mechanical properties, GO is commonly used to reinforce natural polymers with lower structural stability, such as collagen and gelatin [16,19]. Beyond providing structural support, GO plays a biological role by promoting key cellular functions essential to tissue regeneration, including adhesion, proliferation, and differentiation [19,20,21]. Furthermore, GO demonstrates osteoinductive potential both as a surface coating [22] and when incorporated into composites for bone regeneration therapies [19,21].

To enhance the osteogenic capacity of gelatin-based hydrogels, calcium phosphate-based bioceramics such as hydroxyapatite (HA) have been incorporated due to their compositional similarity to the mineral phase of natural bone, as well as their biocompatibility and affinity for polymers [15]. The addition of HA not only increases the bioactivity and osteoconductivity of the hydrogel but also replicates the structure of the natural extracellular matrix, facilitating tissue integration and promoting new bone formation [23]. Moreover, the combination of HA with gelatin has been widely reported to positively influence cell proliferation [24,25,26,27] and increase alkaline phosphatase (ALP) activity [24,27].

Incorporation of natural compounds into composite hydrogels can significantly enhance their bioactive properties [28,29]. Polyphenols, such as tannic acid (TA), are abundant and well-known for their antioxidant [30], anti-inflammatory, and antimicrobial properties [31,32]. In hydrogel systems, TA acts as a physical crosslinker by forming stable hydrogen bonds with gelatin, which improves the structural integrity, enzymatic resistance [33,34], and controlled release of hydrogel components [35]. Additionally, TA contributes to the stabilization of GO [36,37] and forms stable interactions with HA [38]. However, high concentrations of TA may induce cytotoxicity or result in overly rigid crosslinking, potentially compromising scaffold performance. Therefore, the optimization of TA concentration is essential for its safe and effective application in regenerative contexts [35]. Notably, TA has been demonstrated to promote mineralization [39] and proliferation of bone marrow stem cells (BMSCs) by inhibiting reactive oxygen species (ROS) [40], thereby positively influencing bone remodeling and highlighting its relevance in bone regeneration strategies.

This study investigated the physicochemical and mechanical properties of a TA cross-linked gelatin-based hydrogel functionalized with GO and HA. Additionally, the influence on cellular behavior in vitro was assessed using a mouse fibroblast cell line (L929 cells) and canine periodontal ligament-derived cells. Cellular morphology, metabolic activity, and alkaline phosphatase production were also analyzed to assess the potential of the tested gelatin-based hydrogel to be used to develop new bone regeneration therapies.

## 2. Results and Discussion

### 2.1. Physicochemical Characterization of Hydrogels

**Figure 1:**
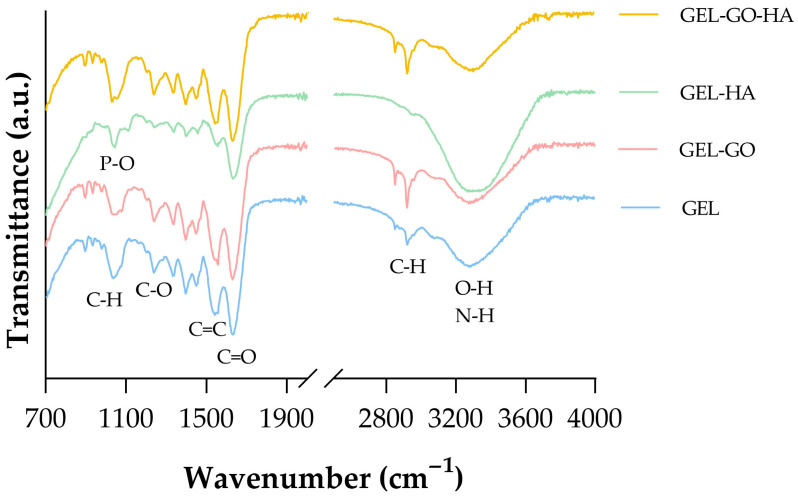
ATR-FTIR spectra of developed TA–gelatin-based hydrogels.

The chemical structure and molecular interactions of Gel (gelatin/TA), Gel-GO, Gel-HA, and Gel-GO-HA hydrogels were characterized using attenuated total reflectance Fourier-transform infrared spectroscopy (ATR-FTIR). The FTIR spectra of all hydrogel formulations are presented in Figure 1. Characteristic absorption bands of gelatin are observed at 3551 cm^−1^, 2932 cm^−1^, 1693 cm^−1^, and 1530 cm^−1^, which corresponds to N–H and O–H stretching vibrations, C–H stretching, C=O stretching (amide I), and N–H bending (amide II), respectively [41,42]. The presence of TA is confirmed by the broad absorption band around 3423 cm^−1^, which is attributed to the stretching vibrations of multiple O–H groups within its polyphenolic structure. Additional prominent peaks associated with TA include esteric C–O stretching at 1693 cm^−1^, which overlaps with gelatin’s amide I band, as well as aromatic C=C stretching at 1606 and 1500 cm^−1^, and C–O stretching at 1205 cm^−1^. Furthermore, out-of-plane aromatic C–H bending vibrations characteristic of TA are evident in the 1000–1300 cm^−1^ region of the hydrogel spectra [41,42]. The characteristic P–O stretching vibration of HA is observed at 1032 cm^−1^ in the FTIR spectrum of the Gel-HA hydrogel [43]. Interestingly, the characteristic absorption bands of GO were not clearly identified in the FTIR spectra of either the Gel-GO or Gel-GO-HA hydrogels. This absence may be attributed to the overlap of GO peaks with those of gelatin or their relatively low relative intensity. Moreover, no new peaks or significant shifts were detected, indicating that no covalent chemical reactions occurred during hydrogel preparation. This suggests that the network formation between gelatin, HA, GO, and TA is governed by non-covalent interactions such as hydrogen bonding.

**Figure 2:**
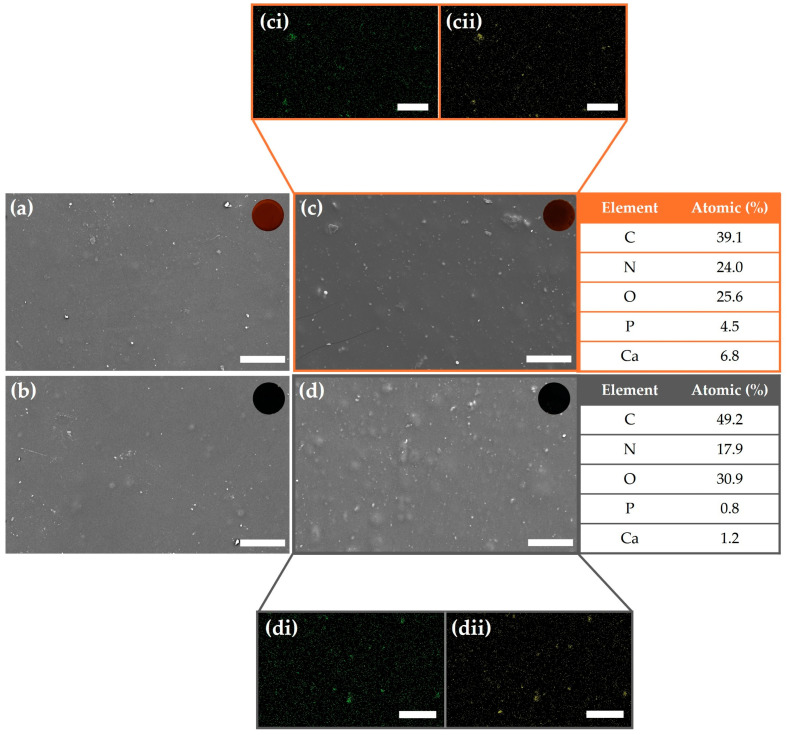
SEM micrographs of hydrogel surfaces: (**a**) Gel, (**b**) Gel-GO, (**c**) Gel-HA, and (**d**) Gel-GO-HA; insets: optical images. EDS elemental maps for phosphorus (**ci**,**di**) and calcium (**cii**,**dii**) are shown for Gel-HA (**c**) and Gel-GO-HA (**d**), respectively. Bar = 40 µm.

Scanning electron microscopy (SEM) was employed to examine the surface morphology and microstructure of the hydrogels, as well as to evaluate the distribution of GO and HA nanoparticles within the TA–gelatin-based matrix (Figure 2). The surface characteristics of hydrogels are crucial in determining water and nutrient permeability and significantly influence cellular behavior such as adhesion, spreading, and proliferation [44].

In the SEM images, scattered bright spots were observed both in Gel-HA (Figure 2c) and Gel-GO-HA (Figure 2d), which correspond to HA nanoparticles. This observation was confirmed by the presence of phosphorus (Figure 2ci,di) and calcium (Figure 2cii,dii) signals in the corresponding energy-dispersive X-ray spectroscopy (EDS) maps, indicating a favorable dispersion of HA within the hydrogel matrix. However, the presence of GO in GeL-GO (Figure 2b) and GeL-GO-HA (Figure 2d) could not be definitively confirmed by SEM/EDS, as the carbon signal may have originated from either the gelatin matrix or GO itself.

The seemingly uniform distribution of nanoparticles within the hydrogels can be attributed to favorable non-covalent interactions, such as hydrogen bonding between the TA–gelatin network and the incorporated nanomaterials. Furthermore, the inset optical images in Figure 2 show the formation of denser and interconnected network structures in the nanoparticle-loaded hydrogels, particularly in the presence of both HA and GO, indicating a synergistic enhancement in the structural integrity of the composite system. Additionally, Figure 2a reveals that the control hydrogel (Gel) exhibited a relatively smooth surface, while the incorporation of nanoparticles in Gel-GO (Figure 2b), Gel-HA (Figure 2c), and Gel-GO-HA (Figure 2d) resulted in visibly rougher textures. This increase in surface roughness is known to promote initial cell attachment, suggesting that the inclusion of GO and HA nanoparticles may enhance the hydrogel’s biointeractivity and cell compatibility [45].

The degradation behavior of TA–gelatin-based hydrogels is significantly affected by their post-production drying times. Drying time serves as a crucial parameter that influences hydrogel properties, including internal microstructure, mechanical integrity, and residual water content. Insufficient drying can result in structurally weak hydrogels with diminished stability and increased susceptibility to environmental interactions. In preliminary studies, extending the drying time resulted in a noticeable decrease in degradation rates across all hydrogels, with the most substantial improvements observed in hydrogels dried for 15 days. This trend aligns with findings from other studies, indicating that extended drying enhances cross-linking density, thereby improving structural integrity and playing pivotal roles in reducing degradation rate [46]. The incorporation of HA into hydrogels (Gel-HA and Gel-GO-HA) significantly decreased the degradation rate, particularly in hydrogels exposed to prolonged drying periods. This phenomenon is attributed to the calcium ions in HA, which facilitates molecular interactions with the hydroxyl and amine groups in gelatin. As a result, these interactions help to stabilize the hydrogel network and diminish water absorption capacity [47].

**Figure 3:**
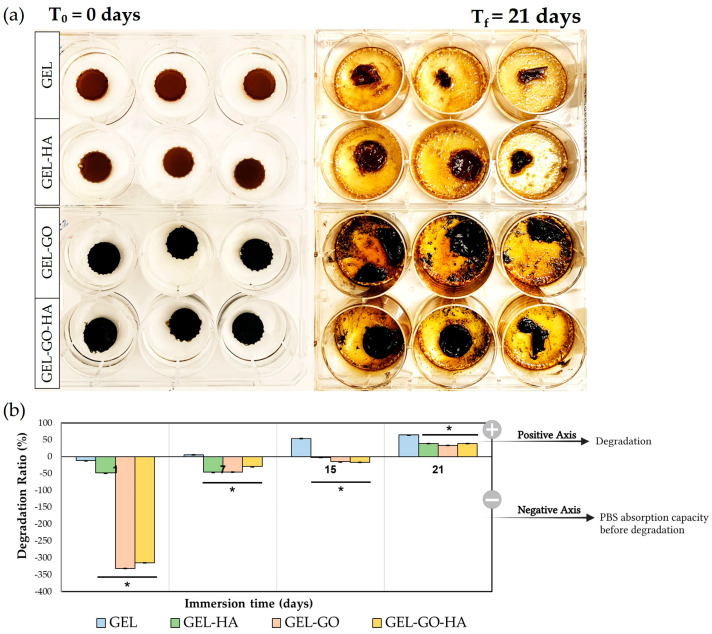
Degradation behavior of TA–gelatin-based hydrogels dried for 15 days and subsequently immersed in PBS solution for 1, 7, 15, and 21 days. (**a**) Representative visual appearance of Gel, Gel-HA, Gel-GO, and Gel-GO-HA hydrogels at the initial time point (T_0_) and after 21 days of immersion (Tf). (**b**) Quantitative degradation ratio (%) of the hydrogels over time. * Significantly different from Gel (*p* ≤ 0.05).

Figure 3 shows the results concerning the hydrogels dried for 15 days that were immersed in 5 mL of phosphate-buffered saline (PBS) at 37 °C to evaluate their degradation profiles over a period of 21 days. Figure 3a illustrates the morphological changes observed in the hydrogels from day 0 to day 21 immersion in PBS. By the end of the 21-day period, most hydrogels exhibited substantial degradation, with the exception of those containing HA nanoparticles (Gel-HA and Gel-GO-HA), which maintained their macroscopic shape despite partial mass loss. The degradation rate of hydrogels is crucial for applications involving in vitro culture media and in vivo body fluids, as it affects the preservation of nutrients and overall scaffold stability. The degradation profiles of the hydrogels over periods of 1, 7, 15, and 21 days are shown in Figure 3b. Hydrogels that were immersed for only 1 day exhibited high absorption rates, particularly those containing GO (Gel-GO and Gel-GO-HA). It became clear that all hydrogels exhibit degradation after 21 days of immersion, regardless of their compositions. However, the extent of degradation was significantly influenced by the presence of reinforcing nanoparticles such as GO and HA. Overall, the results suggest that optimizing the drying time and incorporating nanoparticles like GO and HA are effective strategies to modulate the degradation behavior of TA–gelatin-based hydrogels.

**Figure 4:**
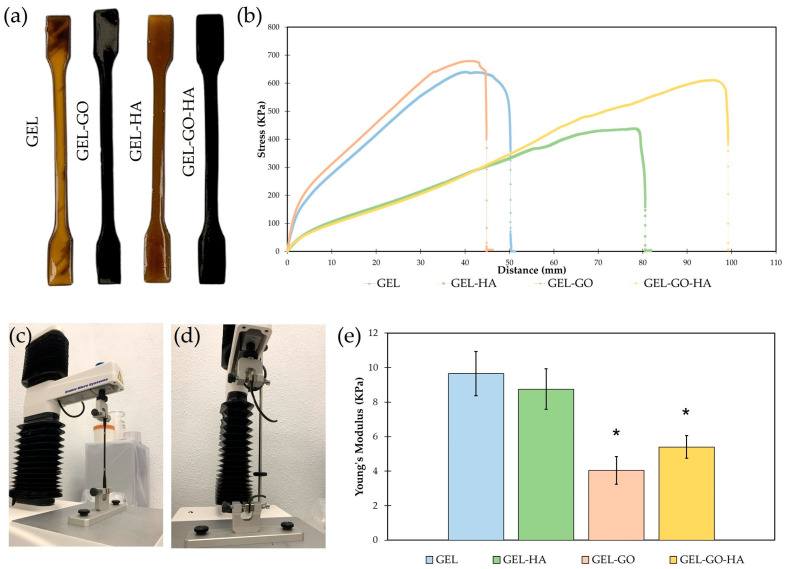
Tensile testing. (**a**) Hydrogel (Gel, Gel-HA, Gel-GO, and Gel-GO-HA) after their production. (**b**) Stress–distance curves representative of all the hydrogels. (**c**,**d**) The tensile test before and after fracture. (**e**) Young’s Modulus of all developed hydrogels. * Significantly different from Gel (*p* ≤ 0.05).

Tensile testing was performed to assess the mechanical performance of the hydrogels. As shown in Figure 4a, the hydrogels display notable visual differences: those containing GO (Gel-GO and Gel-GO-HA) appear dark due to the addition of GO, whereas the control hydrogels (Gel) and with incorporated HA (Gel-HA) display a brown coloration.

The stress–distance curves (Figure 4b) indicate that Gel-GO exhibits the highest peak stress at approximately 720 kPa, demonstrating superior tensile strength. This mechanical reinforcement aligns with existing literature, which suggests that GO enhances intermolecular bonding and load transfer within polymeric matrices due to its large surface area and functional groups [48,49]. In contrast, Gel and Gel-HA demonstrate lower peak stresses, ~650 kPa and ~430 kPa, respectively. The relatively moderate strength of Gel reflects the inherent mechanical limitations of gelatin hydrogels [15], while the reduced strength of Gel-HA suggests that HA alone provides limited reinforcement, likely due to poor particle dispersion or weak interfacial bonding [50]. However, Gel-GO-HA displays the highest elongation at break, measuring about 100 mm, which suggests enhanced ductility and toughness. This phenomenon can be attributed to a synergistic effect, whereas GO contributes to matrix reinforcement, while HA promotes energy dissipation and network flexibility [51]. Gel-HA also exhibits considerable ductility at approximately 80 mm, supporting prior findings that HA can improve deformability by acting as a physical spacer within the network [51,52]. Conversely, Gel and Gel-GO fracture at shorter extensions of about 50 mm and 45 mm, respectively, indicating a more brittle response. This brittleness is particularly pronounced in GO-rich networks, where excessive GO content may lead to aggregation or over-stiffening [48].

The area under the stress–distance curve for Gel-GO-HA indicates the highest toughness, demonstrating its ability to absorb more energy before failure (Figure 4c,d). This enhanced mechanical resilience reflects the synergistic interactions between GO and HA, which contribute to the formation of a denser and more interconnected polymer network [51,52]. While Gel-GO achieves the highest peak stress, its rapid fracture denotes a strong yet brittle profile, a common trait found in nanofiller-reinforced systems [49]. In contrast, Gel-HA, although exhibiting lower strength, demonstrates greater extensibility, supporting the observations that HA enhances flexibility while providing only modest tensile reinforcement [53,54].

The Young’s modulus values presented in Figure 4e support the previously discussed mechanical behavior. The Gel (9.6 ± 1.2 kPa) and Gel-HA (8.5 ± 1.1 kPa) exhibit higher stiffness, as indicated by their higher modulus values. In contrast, GEL-GO (3.8 ± 1.0 kPa) and Gel-GO-HA (5.3 ± 0.8 kPa) demonstrate increased compliance, which can be attributed to the altered polymer network dynamics resulting from the incorporation and distribution of GO and HA nanoparticles. These fillers can disrupt the uniformity of the gelatin matrix, potentially decreasing cross-linking density and enhancing flexibility.

After undergoing tensile deformation, the TA–gelatin-based hydrogels reinforced with GO and HA (Gel-GO-HA) exhibited a clear shape memory behavior, as demonstrated in the Supplementary Materials (Supplementary Video S1). This shape memory behavior is driven by reversible physical crosslinking mechanisms, primarily involving hydrogen bonding, π–π interactions, and ionic coordination. During tensile deformation, the hydrogel’s polymer chains become aligned, leading to a partial disruption of reversible bonds, such as hydrogen bonds between gelatin, TA, and GO. This process enables the hydrogel to maintain a deformed configuration. The presence of GO contributes to this mechanism by providing high-surface-area nanosheets that facilitate dynamic hydrogen bonds and π–π stacking interactions with TA and gelatin, enhancing mechanical integrity and shape retention [49,55]. Meanwhile, HA introduces Ca^2+^-mediated ionic interactions with the carboxyl and amine groups of gelatin, further stabilizing the deformed network structure [56].

### 2.2. Cell Response to Tannic–Gelatin-Based Hydrogels

L929 fibroblasts were first used for an initial cytotoxicity screening, with exposure to various concentrations of gelatin-based hydrogel extracts assessed at 24 and 48 h. This model helped identify the most cytocompatible concentrations by comparison with basal control (without hydrogel extract). Based on these results, a biomimetic canine periodontal ligament (PDL)-derived cell culture model previously described [57] was used to evaluate, over up to 14 days, the cellular response to the incorporation of graphene oxide (GO) and hydroxyapatite (HA) into the hydrogels. The response of PDL-derived cell cultures was analyzed regarding cell viability and proliferation, morphology, and expression of an osteogenic marker (alkaline phosphatase).

#### 2.2.1. Cytotoxicity

**Figure 5:**
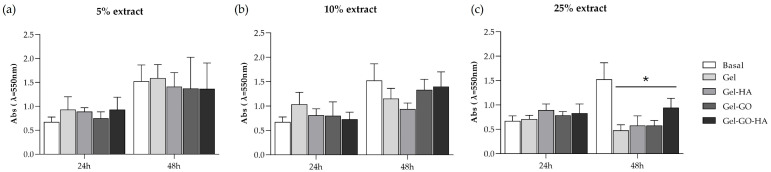
Metabolic activity of L929 cell cultures in basal medium (control) and exposed to 5% (**a**), 10% (**b**) and 25% (**c**) extract of tannic–gelatin-based hydrogel (Gel), Gel with hydroxyapatite 2% (Gel-HA), Gel with graphene oxide 7.5% (Gel-GO), and Gel with graphene oxide 7.5% and hydroxyapatite 2% (Gel-GO-HA) for 24 and 48 h. * Significantly different from the cultures performed in basal conditions (*p* ≤ 0.05).

The L929 cell line is usually used to test the cytocompatibility of materials due to being a representative model of fibroblastic behavior [58]. Metabolic activity was assessed through the MTT assay 24 and 48 h after exposure of the L929 cultures to basal medium (control), and 5% (Figure 5a), 10% (Figure 5b), and 25% (Figure 5c) extracts from tannic–gelatin-based hydrogel (Gel), Gel with hydroxyapatite 2% (Gel-HA), Gel with graphene oxide 7.5% (Gel-GO), and Gel with graphene oxide 7.5% and hydroxyapatite 2% (Gel-GO-HA). According to the ISO 10993-5 standard, materials are considered non-toxic if they yield a cell viability greater than 70%, as determined by the MTT assay [59]. Compared to the control (basal medium), the metabolic activity of cultures exposed to the hydrogel’s extracts (5%, 10%, and 25%) for 24 h showed a tendency to increase. After 48 h, compared to control, exposure to the 5% extracts resulted in similar activity, whereas the presence of the 10% extracts caused a tendency for decreased values for the GEL and GEL-HA hydrogels, although without attaining statistical differences; in contrast, cultures exposed to 25% extracts showed decreased metabolic activity. In summary, results indicated a time- and concentration-dependent cytotoxic effect of the hydrogels’ extracts, however highlighting favorable conditions for cell proliferation at lower concentrations (5% and 10%). These findings (particularly with the high extract concentrations) can be explained by the limitations of in vitro studies, which occur in a closed system and only reflect the short-term cellular response to the hydrogels’ extracts without the complexity, continuous diffusion, and degradation capacity of a more complex in vivo model [60].

#### 2.2.2. Metabolic Activity and Morphology of Canine PDL-Derived Cells

In the next phase, the response of canine PDL-derived cells to extracts from tannic–gelatin-based hydrogels was evaluated, focusing on cell viability/proliferation and morphology.

**Figure 6:**
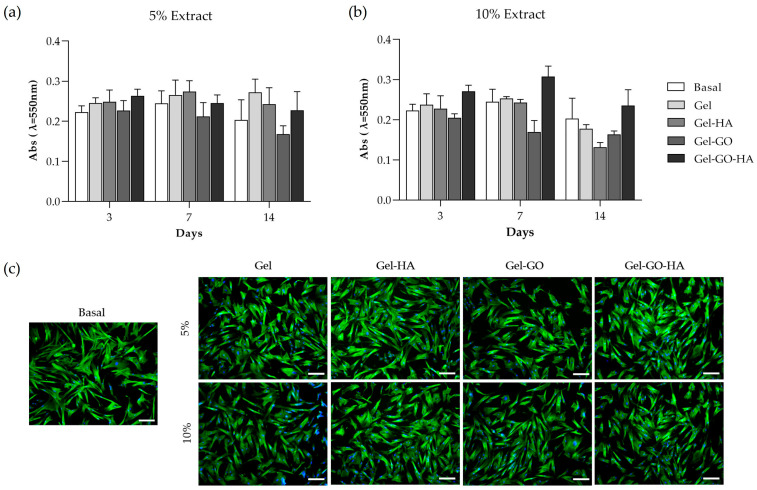
(**a**,**b**) Metabolic activity on days 3, 7, and 14 and (**c**) immunocytochemical staining of F-actin cytoskeleton (green) and nucleus (blue) on day 3 of canine periodontal ligament-derived cell cultures in basal medium (control) and exposed to 5% and 10% extracts of tannic–gelatin-based hydrogel (Gel), Gel with hydroxyapatite 2% (Gel-HA), Gel with graphene oxide 7.5% (Gel-GO), and Gel with graphene oxide 7.5% and hydroxyapatite 2% (Gel-GO-HA). Significant differences were not observed compared to cultures performed in basal conditions. Bar = 100 µm.

The periodontal ligament forms the interface between the alveolar bone and the tooth, being essential to study the cellular response to hydrogels that directly contact this periodontal tissue during guided tissue regeneration therapy [61]. Therefore, canine PDL-derived cell cultures were exposed to 5% (Figure 6a) and 10% (Figure 6b) extracts of Gel, Gel-HA, Gel-GO, and Gel-GO-HA hydrogels to assess their cytocompatibility throughout 14 days of exposure. Metabolic activity was assessed by the MTT assay, and the tested conditions were compared to cultures grown in basal medium. Interestingly, while primary cultures are usually more sensitive to cytotoxic effects [62], on day 3, cultures exposed to both concentrations of hydrogel extracts exhibited similar metabolic activity to control, with no significant toxic effects being observed. The potential cytotoxic effect was only observed at 7-day exposure, with metabolic activity (Figure 6b) showing a tendency to decrease in cultures exposed to Gel-GO extracts and on day 14 for Gel-HA extracts at a concentration of 10%, compared to control, translating into a potential time- and concentration-dependent cytotoxic effect. The slight cytotoxicity effect is likely due to the accumulation of toxic components at higher extract concentrations [60]. Nevertheless, it is interesting to note that the incorporation of GO and HA in the gelatin-based hydrogel (Gel-GO-HA) resulted in a tendency for increased metabolic activity, especially in the presence of the 10% extracts.

Immunofluorescence images of canine PDL-derived cell cultures, 3 days after exposure to the hydrogels’ extracts (Figure 6c), displayed a morphology comparable to the cultures grown in basal medium. Cells presented a spindle-like shape, characteristic of fibroblasts, with nuclei (blue) present and cell-to-cell contact through the filaments of F-actin (green). Qualitative analyses of cellular morphology present a key aspect when evaluating cytotoxicity, allowing for a more reliable assessment of the effects on the cytoskeleton [63]. Cellular response to the extracts of Gel-GO-HA showed promising results with apparent increased proliferation compared to the cultures grown in basal medium, in line with that seen in the MTT assay. Both GO and HA support cell adhesion, proliferation, and differentiation [19,21,24,25,26,27], which may explain the enhanced cellular response observed in the Gel-GO-HA condition.

#### 2.2.3. Osteogenic Response of Canine PDL-Derived Cells

**Figure 7:**
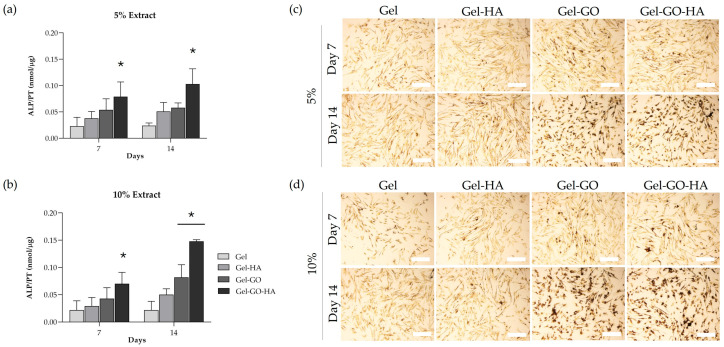
(**a**,**b**) ALP activity and (**c**,**d**) ALP histochemical staining of canine periodontal ligament-derived cell cultures exposed to 5% (**a**) and 10% (**b**) extracts of tannic–gelatin (Gel; control), gel with hydroxyapatite 2% (Gel-HA), gel with graphene oxide 7.5% (Gel-GO), and gel with graphene oxide 7.5% and hydroxyapatite 2% (Gel-GO-HA) for 7 and 14 days. * Significantly different from the cultures exposed to the Gel extract (*p* ≤ 0.05). Bar = 500 µm.

Production of alkaline phosphatase (ALP), a key enzyme marker of early-stage osteogenic differentiation [64], was assessed biochemically on days 7 and 14 for canine–PDL cell cultures exposed to 5% (Figure 7a) and 10% (Figure 7b) extracts of tannic–gelatin-based hydrogels. Cultures exposed to tannic–gelatin-based hydrogel (Gel) extracts were considered as control. ALP activity showed a tendency to increase throughout the days of exposure to hydrogels’ extracts, being higher on day 14. For this exposure time, values were increased in the cultures exposed to the 10% extracts. Cultures exposed to Gel-GO-HA extracts demonstrated increased ALP production consistently in both concentrations and time points compared to the control cultures. These results were corroborated by the ALP histochemical staining images (Figure 7c,d). The presence of ALP is characterized by the brown-to-black staining that increases in a time- and concentration-dependent manner. The osteogenic potential of the incorporated components has been demonstrated with GO being used in bone regeneration therapies [19,21,22] and HA, a natural constituent of bone, promoting osteoconductivity [15,23]. Notably, the combination of GO and HA with natural polymers (gelatin, gelatin–alginate) has also been previously reported to significantly increase ALP activity [16,24,27]. In the present study, the incorporation of the two components, yielding the Gel-GO-HA composite, resulted in a synergistic effect on the osteogenic potential of the hydrogel.

## 3. Conclusions

The addition of polyphenols like tannic acid to gelatin-based hydrogels enhanced crosslinking and biocompatibility of the formulation while reducing the use of synthetic crosslinkers. Using a natural crosslinker allowed for the addition of graphene oxide (GO) and hydroxyapatite (HA) to improve the material’s mechanical strength, biocompatibility, and biofunctionality. The incorporation of both GO and HA demonstrated improved surface roughness, reduced degradation rate, shape memory behavior, increased cell proliferation, and alkaline phosphatase production, all critical properties to produce effective GTR membranes.

These promising results provide a foundation for further investigation, with in vitro studies serving as an initial step to characterize the composite’s behavior across different formulations. Although in vitro models do not fully replicate in vivo conditions, they are appropriate for early-stage development and comply with the 3Rs policy, particularly the reduction and replacement of animal use. Altogether, these findings support the potential of tannic–gelatin-based hydrogels functionalized with GO and HA for the development of new GTR membranes and their evaluation in more representative biological models.

## 4. Materials and Methods

### 4.1. Materials

The following materials were used: Gelatin type B (Mw 40–90 kDa; Acopharma, Barcelona, Spain), Glycerin (80 wt% pure; Lacrilar, Lisbon, Portugal), Polyvinyl alcohol (PVA, Mw 20,000–30,000 g/mol; a gift from Edol, Lisbon, Portugal), Betaine anhydrous (T.C.I. Europe, Zwijndrecht, Belgium), Graphene Oxide (GO, >95 wt% pure, 90% GO particles < 5–7 μm, 50% < 2–4 μm, and 10% < 1–2 μm, reflecting controlled distribution of lateral particle sizes; Graphenea, San Sebastian, Spain), Tannic acid (TA, Mw 1701.20 g/mol; C_76_H_52_O_46_) and ammonia solution (NH_4_OH, 25%) (Sigma-Aldrich, St. Louis, MO, USA), Citric acid monohydrate (C_6_H_8_O_7_·H_2_O, 99.5%) and calcium nitrate tetrahydrate ((Ca(NO_3_)_2_·4H_2_O), 99%) (Riedel-deHaën, Seelze, Germany), and Ammonium hydrogen phosphate ((NH_4_)_2_·HPO_4_, 99%; Merck, Darmstadt, Germany).

### 4.2. Production of Hydroxyapatite Nanoparticles

Hydroxyapatite nanoparticles (HA) were synthesized using the hydrothermal method described by C. Santos et al. [43]. A 0.6 M aqueous solution of citric acid monohydrate was prepared, and its pH was adjusted to 8.1 by adding ammonia solution. Subsequently, a 0.2 M solution of ammonium hydrogen phosphate and a 0.2 M aqueous solution of calcium nitrate tetrahydrate were added to the citric acid solution. The resulting mixture was transferred to a 100 mL Teflon-lined stainless-steel autoclave, which was then sealed and heated at 180 °C for 24 h. The resulting hydroxyapatite precipitate was washed thoroughly with deionized water, filtered through a 0.22 µm membrane filter (Sarstedt, Nümbrecht, Germany), and dried in a desiccator. Nanoparticles demonstrated a monomodal size distribution with an average length of 100–120 nm [43].

### 4.3. Preparation of Tannic Acid–Gelatin-Based Hydrogels’

The control TA–gelatin-based hydrogel was prepared by mixing 30% gelatin and selected excipients with deionized water, using an AHN myLab Analogue Magnetic Stirrer Hotplate (Nordhausen, Germany) at a low shear rate. Initially, glycerin was blended with 80% of the total deionized water and heated to 75 °C. Once this temperature was reached, gelatin and PVA were added to the mixture, with continuous magnetic stirring until complete dissolution. The pH was then adjusted to 8. TA, pre-dissolved in the remaining 20% of deionized water, was added dropwise to the mixture to stabilize while the pH was adjusted with NaOH. The hydrogel was then maintained at 75 °C for one hour with continuous magnetic stirring. Subsequently, betaine was added, and the hydrogel was transferred into cylindrical molds.

In addition to the control formulation, three modified hydrogels were developed by incorporating excipients into the base formulation, specifically, 7.5% GO (GO) and 2% HA nanoparticles. These hydrogels followed the same preparation protocol as the control, with both GO and HA nanoparticles being added at the initial mixing stage. The composition of all hydrogel formulations is summarized in Table 1.

### 4.4. Characterization of Tannic–Gelatin-Based Hydrogels

#### 4.4.1. ATR-FTIR Spectroscopy

To identify the functional groups present in the developed hydrogels, attenuated total reflectance Fourier-transform infrared (ATR-FTIR) spectroscopy was performed using a Nicolet FTIR spectrometer (Thermo Electron, Thermo Fisher Scientific, Waltham, MA, USA). The tannic–gelatin-based hydrogels and hydroxyapatite (HA) nanoparticles were placed directly onto the ATR diamond crystal. Spectra were collected over a wavenumber range of 500 to 4500 cm^−1^ with a resolution of 8 cm^−1^.

#### 4.4.2. Scanning Electron Microscopy

The surface morphology of all dried hydrogels was analyzed using a scanning electron microscope (Phenom ProX G6, Thermo Fisher Scientific, Waltham, MA, USA). No additional treatment was applied to the samples prior to imaging. Specimens were mounted directly onto metal sample holders using carbon tape and observed under the SEM. Energy-dispersive X-ray spectroscopy (EDS) was employed to confirm the presence of hydroxyapatite (HA) and to assess the elemental distribution within the gelatin-based hydrogels. EDS data were acquired using a Bruker Esprit energy-dispersive X-ray spectrometer integrated with the SEM system (Billerica, MA, USA).

#### 4.4.3. Degradation Ratio

To evaluate the degradation behavior of tannic–gelatin-based hydrogels, the hydrogels were first dried for 15 days and then immersed in a phosphate-buffered saline (PBS) solution. Each hydrogel (*n* = 3) was initially weighed (*Wi*) and placed in individual wells of a 6-well tissue culture polystyrene (TCPS) plate containing 5 mL of PBS (pH 7.4). The plates were incubated at 37 °C, and the hydrogels were periodically removed at designated time points (1, 7, 15, and 21 days). After gently removing excess surface water, the hydrogels were reweighed (*Wt*).

The degradation ratio at each time point was determined based on the change in hydrogel weight relative to the initial dry weight, using the following Equation (1):

(1) $$Degradation\;Ratio\;\left(\%\right)=\frac{Wi-Wt}{Wi}\;\times 100$$

#### 4.4.4. Tensile Tests

**Figure 8:**
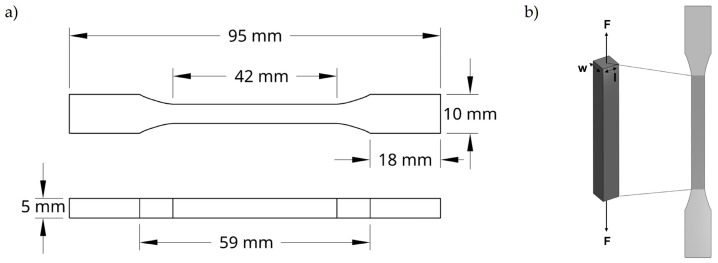
(**a**) Schematic representation of the dog-bone-shaped hydrogel specimen used for tensile testing. (**b**) Illustration of the uniaxial tensile setup, indicating the direction of applied force (F) along the longitudinal axis of the hydrogel.

Uniaxial tensile tests were conducted to evaluate the mechanical properties of the developed hydrogels. These were clamped between two grips set at a fixed initial distance, and the force required to elongate and ultimately break the hydrogels was recorded. Testing was performed in accordance with the ISO 527 standard [65] for specimen geometry, using dog-bone-shaped samples as shown in Figure 8.

Tensile properties were measured using a TA.XTplus Texture Analyser (Stable Micro Systems, Godalming, Surrey, UK) equipped with a 30 kg load cell. The tests were carried out at a constant crosshead speed of 0.5 mm/s. Specimens were securely mounted in the load frame, and tension was applied until failure to assess both the tensile strength and the elongation at break.

### 4.5. Biological Characterization of Tannic–Gelatin-Based Hydrogels

#### 4.5.1. Cell Cultures

L929 cell line (ATCC, Manassas, VA, USA) and dog periodontal ligament (PDL)-derived cells, isolated as described previously [57,66,67], were cultured in alpha-minimum essential medium (α-MEM) supplemented with 10% fetal bovine serum (FBS), 100 IU/mL penicillin, 100 µg/mL streptomycin, and 2.5 µg/mL amphotericin B (All reagents from Gibco, NY, USA). Cultures were incubated at 37 °C in a humidified 5% CO_2_ atmosphere.

For the preparation of the extracts, following the ISO 10993 standard [68], hydrogels (dried for 15 days) were sterilized under UV light for 30 min on each side, incubated in α-MEM supplemented with antibiotics for 24 h and, subsequently, the supernatant was collected and diluted (5%, 10%, and 25%) to be added to the adhered cell cultures.

L929 cells and dog PDL-derived cells were cultured for the evaluation of viability/proliferation, while dog PDL-derived cells were also cultured to assess the biofunctionality of the hydrogels.

#### 4.5.2. Viability/Proliferation (MTT Assay)

For the initial cytotoxicity screening, L929 cells and dog PDL-derived cells were seeded at a density of 10^5^ cells/cm^2^ and 10^4^ cells/cm^2^, respectively, and incubated for 24 h and 48 h, respectively, to allow for adhesion. Afterward, the medium was replaced by fresh medium containing 5%, 10%, or 25% hydrogel extracts in L929 cell cultures and 5% and 10% extracts in dog PDL-derived cell cultures. Cultures were evaluated for viability/proliferation through the MTT assay at specific time points.

MTT [3-(4,5-dimethylthiazol-2-yl)-2,5-diphenyltetrazolium bromide] (Sigma-Aldrich, St. Louis, MO, USA) at a concentration of 5 mg/mL was added to the cultures and incubated at 37 °C for 3 h. After, the medium was removed and the formed formazan salts responsible for the purple color were dissolved with dimethyl sulfoxide (DMSO; Sigma-Aldrich, St. Louis, MO, USA). Absorbance was evaluated at 550 nm using a microplate reader (Synergy HT, Biotek, Winooski, VT, USA).

#### 4.5.3. Total Protein Content and Alkaline Phosphatase Activity

Dog PDL-derived cellular lysates were prepared by incubating cells with 0.1% Triton X-100 (Sigma-Aldrich, St. Louis, MO, USA) for 30 min. These lysates were used to assess total protein content and alkaline phosphatase (ALP) activity.

Total protein content was assessed with the DC^TM^ Protein Assay (BioRad, Hercules, CA, USA) according to the manufacturer’s instructions.

ALP activity was determined by the hydrolysis of 25 mM p-nitrophenyl phosphate (p-NPP) in an alkaline buffer (pH 10.3) at 37 °C for one hour. The reaction was stopped with 5 M NaOH (All reagents from Sigma-Aldrich, St. Louis, MO, USA), and the resulting p-nitrophenol was quantified at 400 nm using a microplate reader (Synergy HT, BioTek, Winooski, VT, USA). ALP activity was normalized to total protein and expressed as nanomoles of p-nitrophenol per microgram of protein (nmol/µg protein).

#### 4.5.4. Alkaline Phosphatase Histochemical Staining Assay

Cell cultures were fixed with 1.5% glutaraldehyde in 0.14 M sodium cacodylate buffer (TAAB, Berks, England) for 15 min and stained for ALP. Fixed cultures were incubated for one hour in a filtered solution of 2 mg/mL sodium naphthyl phosphate and 2 mg/mL Fast Blue RR in 0.1 M Tris buffer (pH 10; Sigma-Aldrich, St. Louis, MO, USA) protected from light. ALP appeared as brown-to-black staining.

#### 4.5.5. Immunocytochemical Staining Assay of F-Actin Cytoskeleton and Nucleus

PDL-derived cell cultures were fixed with 3.7% formaldehyde for 10 min, permeabilized with 0.1% Triton X-100 in PBS for 30 min and blocked with 1% BSA (Bovine serum albumin) in PBS for 30 min (All reagents from Sigma-Aldrich, St. Louis, MO, USA). For F-actin and nuclear staining, cells were incubated with 1:100 Alexa Fluor^®^ 488 phalloidin for 30 min (Molecular Probes, Eugene, OR, USA) and 8 µg/mL Hoechst for 10 min (Enzo, New York, NY, USA). Images were acquired using the Celena S digital imaging system (Logos Biosystems, Anyang-si, Republic of Korea).

### 4.6. Statistical Analysis

Results were collected from three independent experiments, each performed in triplicate, and presented as mean ± standard deviation. Statistical evaluations were carried out using JMP^®^ version 7.0. Differences between experimental conditions were analyzed with the t-test, while comparisons among multiple groups were conducted using one-way ANOVA followed by Tukey’s post hoc test. In all analyses, *p*-values ≤ 0.05 were considered statistically significant.

## Figures and Tables

**Table 1:** Qualitative and quantitative composition of all developed hydrogels.

Hydrogel	Gelatin (%)	Glycerin (%)	PVA (%)	Betaine (%)	TA (%)	GO (%)	HA NPs (%)	H_2_O (%)
TA–Gelatin (Gel)	30	10	1.5	3	0.9	-	-	54.6
Gel-GO	30	10	1.5	3	0.9	7.5	-	47.1
Gel-HA	30	10	1.5	3	0.9	-	2	52.6
Gel-GO-HA	30	10	1.5	3	0.9	7.5	2	45.1

## Data Availability

The original contributions presented in this study are included in the article. Further inquiries can be directed to the corresponding author.

## Supplementary Materials

The following supporting information can be downloaded at: https://www.mdpi.com/article/10.3390/gels11080650/s1, Video S1: TA–gelatin-based hydrogels reinforced with GO and HA (Gel-GO-HA) shape memory behavior after tensile deformation.

## Abbreviations

The following abbreviations are used in this manuscript:

ALP	Alkaline Phosphatase
ATR-FTIR	Attenuated total reflectance Fourier-transform infrared spectroscopy
BSA	Bovine Serum Albumin
DMSO	Dimethyl Sulfoxide
EDS	Energy-dispersive X-ray spectroscopy
FBS	Fetal Bovine Serum
GBR	Guided Bone Regeneration
GO	Graphene Oxide
GTR	Guided Tissue Regeneration
HA	Hydroxyapatite Nanoparticles
MTT	3-(4,5-dimethylthiazol-2-yl)-2,5-diphenyltetrazolium bromide
PBS	Phosphate-buffered Saline
PD	Periodontal Disease
PDL	Periodontal Ligament
p-NPP	p-Nitrophenyl Phosphate
PT	Total Protein Content
SEM	Scanning Electron Microscopy
TA	Tannic Acid

## References

[1] Cunha E., Tavares L., Oliveira M. (2022). Revisiting Periodontal Disease in Dogs: How to Manage This New Old Problem?. Antibiotics.

[2] Bellows J., Berg M.L., Dennis S., Harvey R., Lobprise H.B., Snyder C.J., Stone A.E.S., Van de Wetering A.G. (2019). 2019 AAHA Dental Care Guidelines for Dogs and Cats. J. Am. Anim. Hosp. Assoc..

[3] Wadhawan A., Gowda T.M., Mehta D.S. (2012). Gore-tex(^®^) versus resolut adapt(^®^) GTR membranes with perioglas(^®^) in periodontal regeneration. Contemp. Clin. Dent..

[4] Aprile P., Letourneur D., Simon-Yarza T. (2020). Membranes for Guided Bone Regeneration: A Road from Bench to Bedside. Adv. Healthc. Mater..

[5] Abe G.L., Sasaki J.I., Tsuboi R., Kohno T., Kitagawa H., Imazato S. (2024). Poly(lactic acid/caprolactone) bilayer membrane achieves bone regeneration through a prolonged barrier function. J. Biomed. Mater. Res. B Appl. Biomater..

[6] Pla R., Sanz-Esporrin J., Noguerol F., Vignoletti F., Gamarra P., Sanz M. (2023). A Synthetic Bio-Absorbable Membrane in Guided Bone Regeneration in Dehiscence-Type Defects: An Experimental In Vivo Investigation in Dogs. Bioengineering.

[7] Gawor J.P., Strøm P., Nemec A. (2022). Treatment of Naturally Occurring Periodontitis in Dogs with a New Bio-Absorbable Regenerative Matrix. Front. Vet. Sci..

[8] Wang D., Zhou X., Cao H., Zhang H., Wang D., Guo J., Wang J. (2023). Barrier membranes for periodontal guided bone regeneration: A potential therapeutic strategy. Front. Mater..

[9] Calciolari E., Ravanetti F., Strange A., Mardas N., Bozec L., Cacchioli A., Kostomitsopoulos N., Donos N. (2018). Degradation pattern of a porcine collagen membrane in an in vivo model of guided bone regeneration. J. Periodontal Res..

[10] Rothamel D., Schwarz F., Fienitz T., Smeets R., Dreiseidler T., Ritter L., Happe A., Zöller J. (2012). Biocompatibility and biodegradation of a native porcine pericardium membrane: Results of in vitro and in vivo examinations. Int. J. Oral. Maxillofac. Implant..

[11] Lukin I., Erezuma I., Maeso L., Zarate J., Desimone M.F., Al-Tel T.H., Dolatshahi-Pirouz A., Orive G. (2022). Progress in Gelatin as Biomaterial for Tissue Engineering. Pharmaceutics.

[12] Mogha P., Iyer S., Majumder A. (2023). Extracellular matrix protein gelatin provides higher expansion, reduces size heterogeneity, and maintains cell stiffness in a long-term culture of mesenchymal stem cells. Tissue Cell.

[13] Milano F., Masi A., Madaghiele M., Sannino A., Salvatore L., Gallo N. (2023). Current Trends in Gelatin-Based Drug Delivery Systems. Pharmaceutics.

[14] Tang Y., Tong X., Conrad B., Yang F. (2020). Injectable and in situ crosslinkable gelatin microribbon hydrogels for stem cell delivery and bone regeneration in vivo. Theranostics.

[15] Wu E., Huang L., Shen Y., Wei Z., Li Y., Wang J., Chen Z. (2024). Application of gelatin-based composites in bone tissue engineering. Heliyon.

[16] Purohit S.D., Singh H., Bhaskar R., Yadav I., Bhushan S., Gupta M.K., Kumar A., Mishra N.C. (2020). Fabrication of Graphene Oxide and Nanohydroxyapatite Reinforced Gelatin–Alginate Nanocomposite Scaffold for Bone Tissue Regeneration. Front. Mater..

[17] Zhihui K., Min D. (2022). Application of Graphene Oxide-Based Hydrogels in Bone Tissue Engineering. ACS Biomater. Sci. Eng..

[18] Ryu S.B., Park K.M., Park K.D. (2022). In situ graphene oxide-gelatin hydrogels with enhanced mechanical property for tissue adhesive and regeneration. Biochem. Biophys. Res. Commun..

[19] Hermenean A., Codreanu A., Herman H., Balta C., Rosu M., Mihali C.V., Ivan A., Dinescu S., Ionita M., Costache M. (2017). Chitosan-Graphene Oxide 3D scaffolds as Promising Tools for Bone Regeneration in Critical-Size Mouse Calvarial Defects. Sci. Rep..

[20] Kim J., Choi K.S., Kim Y., Lim K.T., Seonwoo H., Park Y., Kim D.H., Choung P.H., Cho C.S., Kim S.Y. (2013). Bioactive effects of graphene oxide cell culture substratum on structure and function of human adipose-derived stem cells. J. Biomed. Mater. Res. A.

[21] Dinescu S., Ionita M., Pandele A.M., Galateanu B., Iovu H., Ardelean A., Costache M., Hermenean A. (2014). In vitro cytocompatibility evaluation of chitosan/graphene oxide 3D scaffold composites designed for bone tissue engineering. Biomed. Mater. Eng..

[22] Kashte S.B., Kadam S., Maffulli N., Potty A.G., Migliorini F., Gupta A. (2024). Osteoinductive potential of graphene and graphene oxide for bone tissue engineering: A comparative study. J. Orthop. Surg. Res..

[23] Kim H.-W., Song J.-H., Kim H.-E. (2005). Nanofiber Generation of Gelatin–Hydroxyapatite Biomimetics for Guided Tissue Regeneration. Adv. Funct. Mater..

[24] Shahi S., Sharifi S., Khalilov R., Dizaj S.M., Abdolahinia E.D. (2022). Gelatin-hydroxyapatite Fibrous Nanocomposite for Regenerative Dentistry and bone Tissue Engineering. Open Dent. J..

[25] Sharifi S., Samiei M., Abdolahinia E.D., Khalilov R., Shahi S., Dizaj S.M. (2020). Gelatin-hydroxyapatite nano-fibers as promising scaffolds for guided tissue regeneration (GTR): Preparation, assessment of the physicochemical properties and the effect on mesenchymal stem cells. J. Adv. Periodontol. Implant. Dent..

[26] Zhu Y., Chen S., Zhang C., Ikoma T., Guo H., Zhang X., Li X., Chen W. (2021). Novel microsphere-packing synthesis, microstructure, formation mechanism and in vitro biocompatibility of porous gelatin/hydroxyapatite microsphere scaffolds. Ceram. Int..

[27] Lian H., Zhang L., Meng Z. (2018). Biomimetic hydroxyapatite/gelatin composites for bone tissue regeneration: Fabrication, characterization, and osteogenic differentiation in vitro. Mater. Des..

[28] Byun H., Jang G.N., Hong M.-H., Yeo J., Shin H., Kim W.J., Shin H. (2022). Biomimetic anti-inflammatory and osteogenic nanoparticles self-assembled with mineral ions and tannic acid for tissue engineering. Nano Converg..

[29] Luo Y., Lin J., Luo X., Luo G., Zou J., Cai L., He Y., Qiu X., Xu H. (2025). Tannic Acid-Programmed Hydroxyapatite Biomineralization Enables Bilayered Bone-Mimetic Hydrogels for Mandibular Regeneration. Chem. Mater..

[30] Lee H.-Y., Hwang C.-H., Kim H.-E., Jeong S.-H. (2018). Enhancement of bio-stability and mechanical properties of hyaluronic acid hydrogels by tannic acid treatment. Carbohydr. Polym..

[31] Payne D.E., Martin N.R., Parzych K.R., Rickard A.H., Underwood A., Boles B.R. (2013). Tannic acid inhibits *Staphylococcus aureus* surface colonization in an IsaA-dependent manner. Infect. Immun..

[32] Yeo J., Lee J., Yoon S., Kim W.J. (2020). Tannic acid-based nanogel as an efficient anti-inflammatory agent. Biomater. Sci..

[33] Zheng Y., Liang Y., Zhang D., Sun X., Liang L., Li J., Liu Y.N. (2018). Gelatin-Based Hydrogels Blended with Gellan as an Injectable Wound Dressing. ACS Omega.

[34] Chen C., Yang H., Yang X., Ma Q. (2022). Tannic acid: A crosslinker leading to versatile functional polymeric networks: A review. RSC Adv..

[35] Lee J., Kim G. (2018). Calcium-Deficient Hydroxyapatite/Collagen/Platelet-Rich Plasma Scaffold with Controlled Release Function for Hard Tissue Regeneration. ACS Biomater. Sci. Eng..

[36] Yao G., Liu X., Zhang G., Han Z., Liu H. (2021). Green synthesis of tannic acid functionalized graphene hydrogel to efficiently adsorb methylene blue. Colloids Surf. A Physicochem. Eng. Asp..

[37] Luo J., Lai J., Zhang N., Liu Y., Liu R., Liu X. (2016). Tannic Acid Induced Self-Assembly of Three-Dimensional Graphene with Good Adsorption and Antibacterial Properties. ACS Sustain. Chem. Eng..

[38] Anggresani L., Rahayu A., Perawati S., Yulianis Y., Sintia U., Setiawan R. (2024). Synthesis and structure of hydroxyapatite/tannin composites. Polimery.

[39] Kong W., Du Q., Qu Y., Shao C., Chen C., Sun J., Mao C., Tang R., Gu X. (2022). Tannic acid induces dentin biomineralization by crosslinking and surface modification. RSC Adv..

[40] Zhang W., Zhang Y., Li X., Cao Z., Mo Q., Sheng R., Ling C., Chi J., Yao Q., Chen J. (2022). Multifunctional polyphenol-based silk hydrogel alleviates oxidative stress and enhances endogenous regeneration of osteochondral defects. Mater. Today Bio.

[41] Taheri P., Jahanmardi R., Koosha M., Abdi S. (2020). Physical, mechanical and wound healing properties of chitosan/gelatin blend films containing tannic acid and/or bacterial nanocellulose. Int. J. Biol. Macromol..

[42] Kim N.E., Park S., Kim S., Choi J.H., Kim S.E., Choe S.H., Kang T.w., Song J.E., Khang G. (2023). Development of Gelatin-Based Shape-Memory Polymer Scaffolds with Fast Responsive Performance and Enhanced Mechanical Properties for Tissue Engineering Applications. ACS Omega.

[43] Santos C., Gomes P.S., Duarte J.A., Franke R.P., Almeida M.M., Costa M.E., Fernandes M.H. (2012). Relevance of the sterilization-induced effects on the properties of different hydroxyapatite nanoparticles and assessment of the osteoblastic cell response. J. R. Soc. Interface.

[44] Camci-Unal G., Nichol J.W., Bae H., Tekin H., Bischoff J., Khademhosseini A. (2013). Hydrogel surfaces to promote attachment and spreading of endothelial progenitor cells. J. Tissue Eng. Regen. Med..

[45] Yu Z., Xiao C., Huang Y., Chen M., Wei W., Yang X., Zhou H., Bi X., Lu L., Ruan J. (2018). Enhanced bioactivity and osteoinductivity of carboxymethyl chitosan/nanohydroxyapatite/graphene oxide nanocomposites. RSC Adv..

[46] Sreekumaran S., Radhakrishnan A., Rauf A.A., Kurup G.M. (2021). Nanohydroxyapatite incorporated photocrosslinked gelatin methacryloyl/poly(ethylene glycol)diacrylate hydrogel for bone tissue engineering. Prog. Biomater..

[47] Firouzi N., Baradar Khoshfetrat A., Kazemi D. (2020). Enzymatically gellable gelatin improves nano-hydroxyapatite-alginate microcapsule characteristics for modular bone tissue formation. J. Biomed. Mater. Res. Part A.

[48] Shin S.R., Li Y.C., Jang H.L., Khoshakhlagh P., Akbari M., Nasajpour A., Zhang Y.S., Tamayol A., Khademhosseini A. (2016). Graphene-based materials for tissue engineering. Adv. Drug Deliv. Rev..

[49] Yi J., Choe G., Park J., Lee J.Y. (2020). Graphene oxide-incorporated hydrogels for biomedical applications. Polym. J..

[50] Dorozhkin S.V. (2010). Bioceramics of calcium orthophosphates. Biomaterials.

[51] Li M., Xiong P., Yan F., Li S., Ren C., Yin Z., Li A., Li H., Ji X., Zheng Y. (2018). An overview of graphene-based hydroxyapatite composites for orthopedic applications. Bioact. Mater..

[52] Xing W., Tang Y. (2022). On mechanical properties of nanocomposite hydrogels: Searching for superior properties. Nano Mater. Sci..

[53] Kim D., Lee J., Kim G. (2020). Biomimetic gelatin/HA biocomposites with effective elastic properties and 3D-structural flexibility using a 3D-printing process. Addit. Manuf..

[54] Utech S., Boccaccini A.R. (2016). A review of hydrogel-based composites for biomedical applications: Enhancement of hydrogel properties by addition of rigid inorganic fillers. J. Mater. Sci..

[55] Guo J., Sun W., Kim J.P., Lu X., Li Q., Lin M., Mrowczynski O., Rizk E.B., Cheng J., Qian G. (2018). Development of tannin-inspired antimicrobial bioadhesives. Acta Biomater..

[56] Chatterjee S., Bohidar H.B. (2005). Effect of cationic size on gelation temperature and properties of gelatin hydrogels. Int. J. Biol. Macromol..

[57] Pinho L.C., Queirós J.A., Santos C., Colaço B., Fernandes M.H. (2024). Biomimetic In Vitro Model of Canine Periodontal Ligament. Int. J. Mol. Sci..

[58] Wang M.O., Etheridge J.M., Thompson J.A., Vorwald C.E., Dean D., Fisher J.P. (2013). Evaluation of the in vitro cytotoxicity of cross-linked biomaterials. Biomacromolecules.

[59] (2009). Biological Evaluation of Medical Devices—Part 5: Tests for in Vitro Cytotoxicity.

[60] Rosengren A., Faxius L., Tanaka N., Watanabe M., Bjursten L.M. (2005). Comparison of implantation and cytotoxicity testing for initially toxic biomaterials. J. Biomed. Mater. Res. Part A.

[61] Koch F., Meyer N., Valdec S., Jung R.E., Mathes S.H. (2020). Development and application of a 3D periodontal in vitro model for the evaluation of fibrillar biomaterials. BMC Oral Health.

[62] Rosa V., Silikas N., Yu B., Dubey N., Sriram G., Zinelis S., Lima A.F., Bottino M.C., Ferreira J.N., Schmalz G. (2024). Guidance on the assessment of biocompatibility of biomaterials: Fundamentals and testing considerations. Dent. Mater..

[63] Hazen M.J., Freire P.F., Martín J.P., Peropadre A., Herrero Ó., López L., Labrador V. (2010). The importance of microscopic analysis for accurate interpretation of chemical-induced cytotoxicity. Microsc. Sci. Technol. Appl. Educ..

[64] Vimalraj S. (2020). Alkaline phosphatase: Structure, expression and its function in bone mineralization. Gene.

[65] (2019). Plastics—Determination of Tensile Properties—Part 1: General Principles.

[66] Tanaka K., Iwasaki K., Feghali K.E., Komaki M., Ishikawa I., Izumi Y. (2011). Comparison of characteristics of periodontal ligament cells obtained from outgrowth and enzyme-digested culture methods. Arch. Oral. Biol..

[67] Banyatworakul P., Osathanon T., Chumprasert S., Pavasant P., Pirarat N. (2021). Responses of canine periodontal ligament cells to bubaline blood derived platelet rich fibrin in vitro. Sci. Rep..

[68] (2021). Biological Evaluation of Medical Devices—Part 12: Sample Preparation and Reference Materials.

